# Complex management of concurrent lower extremity arteriovenous malformations: A case study and review of interventional techniques

**DOI:** 10.1016/j.radcr.2024.07.197

**Published:** 2024-08-29

**Authors:** Caroline J. Cushman, Andrew F. Ibrahim, James Montgomery

**Affiliations:** a Department of Radiology, Health Sciences Center, School of Medicine, Texas Tech University, Lubbock, TX, USA; b Department of Interventional Radiology, Covenant Medical Center, Lubbock, TX, USA

**Keywords:** Arteriovenous malformations, Lower extremity

## Abstract

Arteriovenous malformations (AVMs) are rare vascular anomalies that present complex diagnostic and therapeutic challenges, particularly in uncommon locations such as the lower extremities. A 72-year-old female with chronic atrial fibrillation, hypertension, and peripheral vascular disease presented with severe lower extremity edema due to multiple AVMs below the knee. This case underscores the importance of a multidisciplinary, individualized approach in managing complex AVMs and highlights the need for advanced imaging and diverse interventional techniques to ensure effective treatment and long-term outcomes.

## Introduction

Arteriovenous malformations (AVMs) are uncommon, aberrant vascular connections between an artery and a vein that bypass the capillary system, forming high-flow vascular masses prone to hemorrhage due to vulnerabilities in their altered vessel wall structure and damage from high-pressure arterial flow against low-pressure venous walls [1,2]. Patients often experience venous hypertension, which can lead to serious complications such as ulcers, pain, and high output congestive heart failure [3]. AVMs are most frequently found in the brain, neck, and pelvis, with occurrences in the lower extremities being rare [4]. When present in the lower extremities, they often cause vague symptoms like nonspecific pain and swelling, leading to frequent misdiagnoses [4]. The specific pathogenesis of AVMs is yet to be elucidated, and diagnosis and treatment can vary depending on lesion location and associated clinical symptoms, complicating their management. Herein, we present a case of a patient with 3 concurrent lower extremity AVMs and the complex interventional treatments used to manage these malformations.

## Case presentation

A 72-year-old female with a complex medical history, including chronic atrial fibrillation and hypertension, presented with moderate lower extremity edema and pain, predominantly in the left leg. Physical examination revealed significant swelling, warmth, and bluish discoloration of the left lower extremity, with palpable thrill and audible bruit over the medial ankle region.

**Fig. 1:**
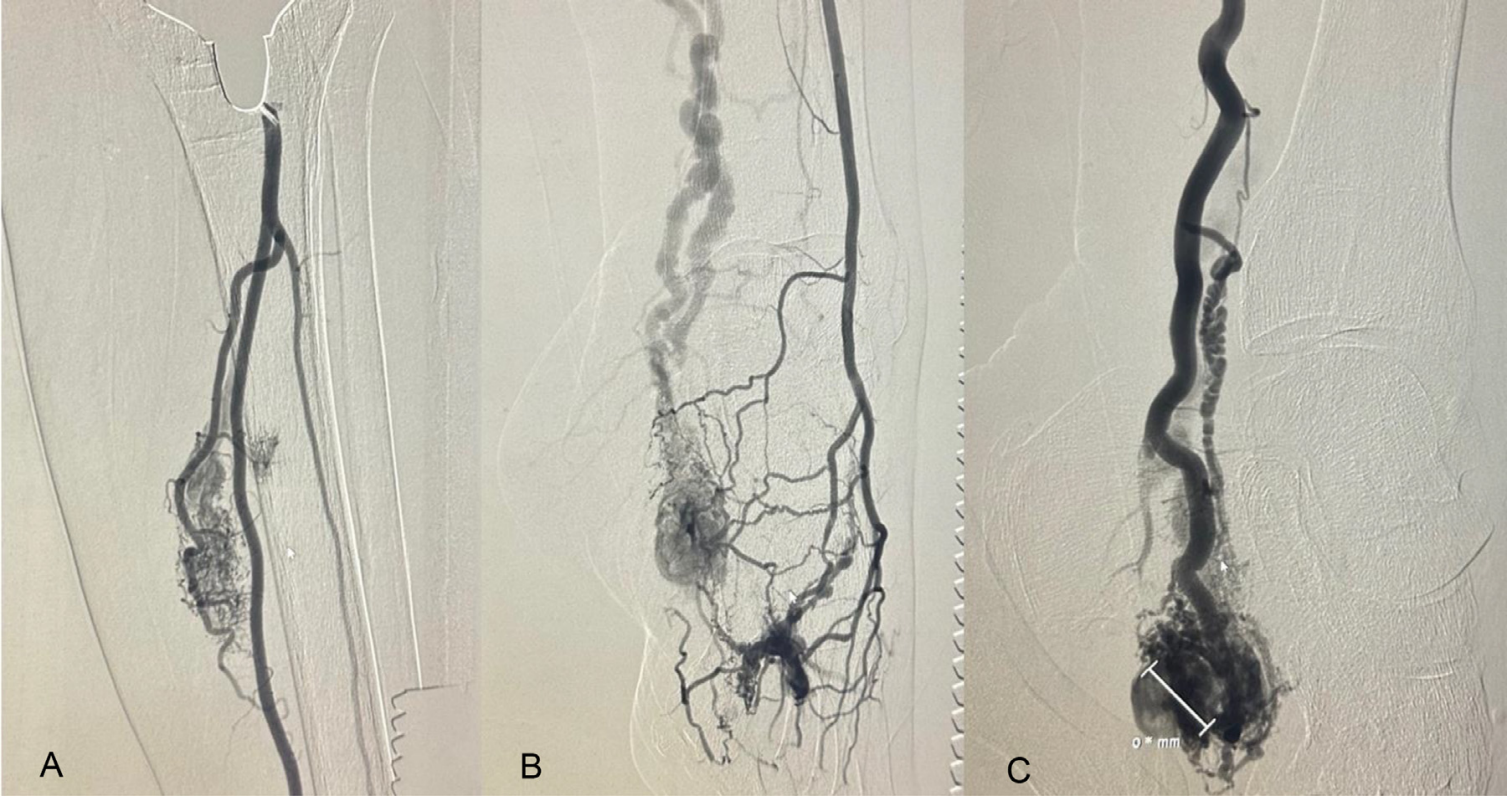
A diagnostic arteriogram revealed arteriovenous malformations (AVMs) below the left knee, identified as 3 distinct AVMs: one originating from the peroneal artery (A), another from the distal anterior tibial artery on the plantar aspect of the foot (B), and a third from the posterior tibial artery (C).

Initial imaging using Doppler ultrasound, CT angiography (CTA), and MRI identified multiple AVMs below the knee on the left side, including a small arteriovenous fistula (AVF) arising from the posterior tibial artery in the calf and several smaller malformations in the foot (Fig. 1). Doppler ultrasound was used to assess blood flow and identify abnormal connections between arteries and veins, while CTA and MRI provided detailed anatomical visualization and helped delineate the extent and structure of the AVMs. A diagnostic arteriogram further characterized these AVMs, revealing 3 distinct malformations: one from the posterior tibial artery, one from the peroneal artery, and one at the plantar aspect of the foot arising from the distal anterior tibial artery. The largest AVM at the medial ankle region was predominantly supplied by the posterior tibial artery, with additional branches from the anterior tibial artery.

Given the multiple feeding vessels and extensive nature of the malformations, Onyx glue was initially selected for embolization of the branches of the posterior tibial artery supplying the AVM. Onyx is an ethylene-vinyl alcohol copolymer dissolved in dimethyl sulfoxide (DMSO). It is a nonadhesive liquid embolic agent that solidifies upon contact with blood, creating a cohesive and durable embolus. Onyx was chosen for its ability to penetrate the complex vascular network of the AVM and provide a complete and more permanent occlusion compared to other agents. Due to the small AVF particles were not a consideration.

Using a microcatheter and fluoroscopic guidance, Onyx glue was carefully injected to occlude the feeding vessels. Fluoroscopy provided real-time imaging, allowing precise navigation of the catheter and controlled delivery of the embolic agent. However, the patient developed significant pain in the medial ankle region postembolization, likely due to ischemia of the posterior tibial nerve. This postsurgical pain was managed with a local injection of the anesthetic Exparel. Following this complication, Onyx glue was not used again. All subsequent treatments utilized particles and coils, including for the large AVM arising from the calf and supplied by the peroneal artery, as well as the AVM in the foot arising from the anterior tibial artery.

The particles used for embolization were polyvinyl alcohol (PVA) particles, which are commonly employed due to their ability to occlude small blood vessels effectively. These particles were selected for their precise size and shape, allowing them to target specific areas of the AVM while minimizing impact on surrounding tissues. Smaller particles were preferred to reduce the risk of recurrence.

Ruby coils, used in the subsequent embolization procedures, were made of platinum. Platinum is chosen because it is biocompatible, visible under fluoroscopy, and has the necessary flexibility and durability for precise deployment in vascular procedures. Coils induce clot formation by creating a physical barrier that slows blood flow and promotes thrombosis, effectively blocking the vessel. They are particularly useful for larger vessels or when precise control over the embolization process is required. The procedures utilized a combination of Ruby coils (1 × 2, 2 × 2, 3 × 7, and 3 × 20 sizes) and 700-900 embospheres.

A month after the initial diagnosis, further arteriograms and embolizations were performed, targeting the large AVF in the medial aspect of the hindfoot. Arteriograms provided detailed images of the blood vessels, allowing for precise identification and targeting of the AVM feeder vessels. Using a combination of a septor balloon catheter and Traxis micro-wire, 0.7 mL and 0.6 mL of Onyx glue was delivered to embolize the distal and proximal portions of the AVF, respectively. The smaller branches feeding this AVM from the anterior tibial artery were embolized with Ruby coils. Additional arteriograms revealed some residual arterial vascularity, necessitating further embolization with Onyx, which achieved near-complete occlusion of the AVF. A directional microcatheter and Traxis wire were then used to access and ablate a third-order vessel supplying the AVM with 1.6 mL of Onyx.

**Fig. 2:**
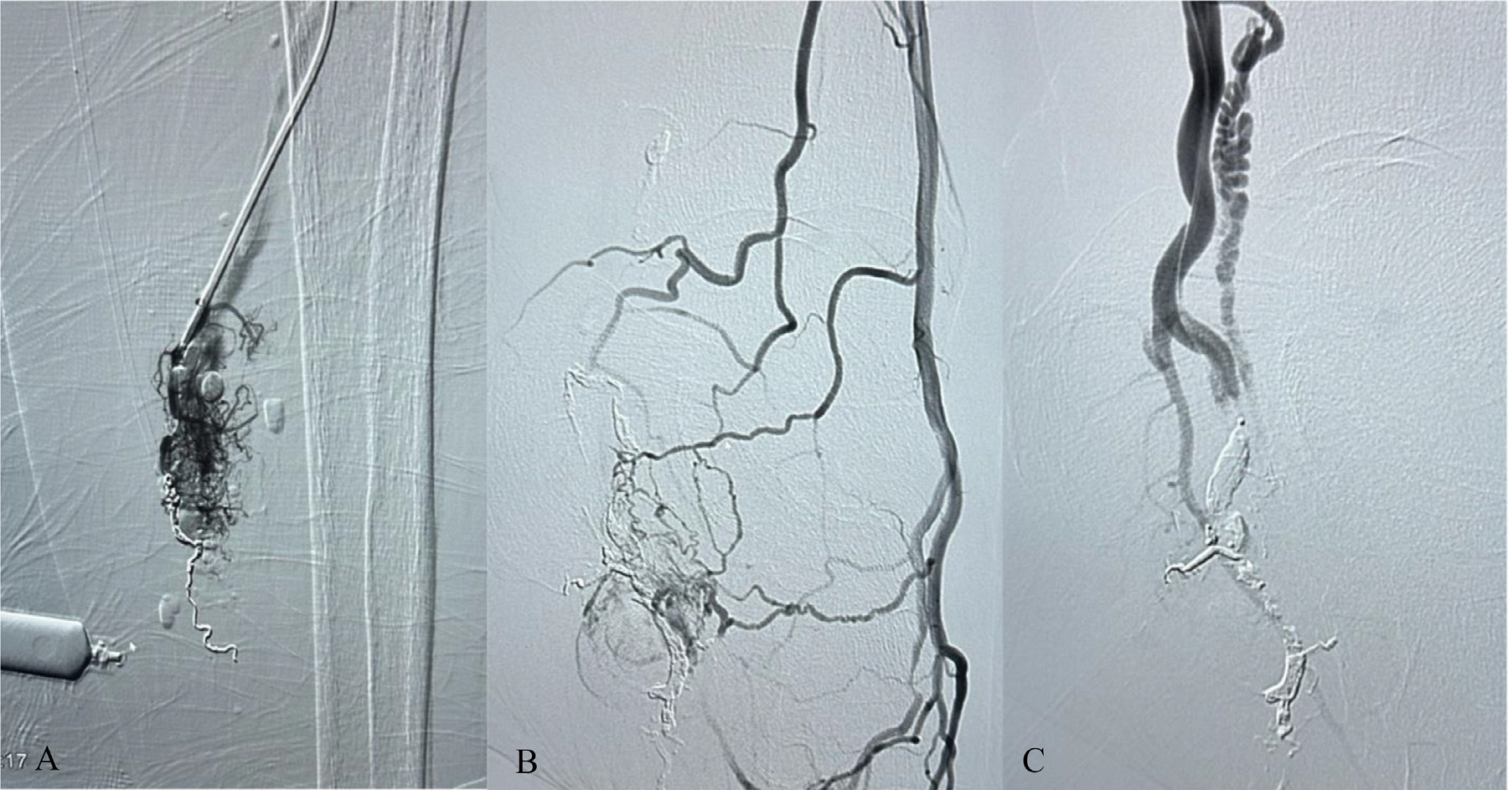
A diagnostic arteriogram revealed arteriovenous malformations (AVMs) which were treated through embolization. The AVMs supplied by the peroneal artery were embolized using Ruby coils (sizes: 1 × 2, 2 × 2, 3 × 7, and 3 × 20) and 700-900 embospheres (A). The AVM in the anterior tibial artery (B) and the posterior tibial artery (C) was embolized with onyx.

A month following the last intervention, another embolization procedure was conducted to treat a large arteriovenous reformation in the right mid-calf, using a combination of Ruby coils and embospheres. The embolization was performed under fluoroscopic guidance, ensuring precise placement of the embolic agents. Multiple arteriograms confirmed near-complete occlusion of the AVM. A final arteriogram of the posterior tibial artery showed no significant arterial flow to the previously treated AVM, indicating successful embolization (Fig. 2).

Follow-up imaging using CT angiography showed some minimal flow supplying the arteriovenous malformations but no significant arterial flow to the treated areas, suggesting effective treatment. Follow-up Doppler ultrasound examinations and annual MRI scans provided ongoing monitoring, allowing for early detection of any potential recurrence. Currently, the patient remains stable with no recurrence of symptoms, and her peripheral vascular disease is being closely monitored with ongoing follow-up care.

## Discussion

The precise etiology of AVMs remains unclear. AVM lesions generally manifest at birth, but the majority do not exhibit clinical symptoms until later in childhood. Their severity can be influenced by hormone-level fluctuations associated with puberty, pregnancy, hormonal treatments, or external factors such as surgical procedures and trauma [5]. While AVMs are frequently found intracranially, extracranial AVMs also commonly appear in the head, neck, trunk, and extremities [6,7]. The exact prevalence of AVMs is still undetermined, though it is estimated to range from approximately 0.89 to 1.34 cases per 100,000 person-years [7].

The clinical presentation of AVMs is highly variable and is influenced by the location of the malformation. To aid clinicians in the systematic diagnosis of vascular disorders, an anatomical classification of peripheral AVMs, based on the primary site of AVM lesions, has been proposed. The classification system is detailed below (Table 1).

**Table 1:** Cho Classification for peripheral arteriovenous malformations (AVMs) [5].

Type of AVMs	Angiographic manifestations
I	Arteriovenous fistula with 3 or less feeding arteries and a single draining vein.
IIa	Multiple arterioles shunt to focal segment of the single draining vein.
IIb	Multiple arterioles shunt to the venous sac with multiple draining veins.
IIc	Multiple arterioles shunt along the long segment of the draining vein.
IIIa	Multiple arterioles shunt to multiple draining veins through multiple fine fistulae.
IIIb	Multiple arterioles shunt to multiple draining veins through multiple enlarged fistulae.

Based off this classification system, the series of AVMs we saw fell under Cho's IIIa.

The symptoms of peripheral arteriovenous malformations (AVMs) vary depending on the size, number of shunts, and the amount of tissue involved [5]. In general, AVMs cause tissue enlargement due to the expansion of arteries and veins, resulting in faster and larger growth of the affected tissue compared to normal tissue.

Initially, most AVMs grow quietly, with the overlying skin appearing reddish and warm [6]. As the AVM progresses, the veins expand, causing noticeable swelling [7]. Our patient presented with severe swelling in the ipsilateral extremity, which aggravated in the standing position due to venous hypertension. Advanced stages of peripheral AVMs can lead to significant tissue damage, manifesting as pain, pressure sores, ulcers, and potential bleeding. Additionally, physical examination often reveals dilated, tortuous veins, increased local skin temperature, persistent palpable tremors, and audible murmurs [8]. Bleeding, ulcers, and infections may occur due to skin and mucosal ischemia, particularly in acral AVMs [8]. In later severe stages, patients, especially those with large shunts, may experience symptoms of congestive heart failure, such as palpitations, shortness of breath, and chest tightness [9]. This decompensated manifestation is typically observed in patients with AVMs in the head, neck, and trunk regions, whereas it occurs in the extremities of patients with extensive and progressive AVMs [5].

Clinical staging of arteriovenous malformations (AVMs) is characterized by assessing flow velocity and the extent of the malformation using Doppler ultrasound, along with the degree of expansion determined by the Schobinger scale [10]. Key differential diagnoses include hamartoma, hemangioma, lymphatic malformation, venous malformation, and capillary malformation [11]. Due to their progressive nature and tendency to recur, treating AVMs often requires a multidisciplinary approach [12,13].

Schobinger's method, popularized by Mulliken et al., [6] is the primary method for clinical staging of AVMs. This method classifies AVMs into 4 stages based on clinical symptoms (Table 2).

**Table 2:** Schobinger's method for clinical staging of AVM's [5].

Stage	Angiographic clinical manifestations
I	Characterized by a bluish stain, warmth, and arteriovenous shunting as revealed via Doppler scanning.
II	Includes all features of Stage I with additional signs of enlargement, pulsations, thrill and bruit, and tortuous/tense veins.
III	Includes all features of Stage II with further complications such as dystrophic skin changes (ulceration, bleeding, persistent pain, or tissue necrosis). Also, known as destruction stage.
IV	Includes all features of Stage III with the addition of congestive cardiac failure.

The angiographic findings in this case are consistent with stage IV AVMs. The patient exhibited clinical manifestations including a bluish discoloration, warmth, and arteriovenous shunting as detected by Doppler scanning. Additional symptoms included enlargement and pulsations of the affected area, presence of thrill and bruit, and tortuous, tense veins. Complications observed were dystrophic skin changes such as ulceration, bleeding, persistent pain, and tissue necrosis.

The gold standard for diagnosing arteriovenous malformations (AVMs) is digital subtraction angiography (DSA) due to its ability to provide detailed images of blood vessels and the vascular structure of the lesion. However, DSA has limitations, particularly in cases involving rapid arteriovenous shunts, where the contrast agent may prematurely enter the refluxing vein, obscuring the malformed vascular mass. Therefore, in our case, we utilized enhanced CT imaging in addition to DSA. Enhanced CT offers clear advantages in displaying the extent of the lesion, blood flow status, feeding arteries, reflux veins, and the relationship between the lesion and adjacent tissues. This comprehensive imaging approach allowed us to establish a more accurate diagnosis and plan for effective treatment.

Treatment of AVMs involves various modalities, each tailored to the specific characteristics of the lesion. The primary treatment strategy is interventional embolization, which aims to eliminate or relieve venous hypertension by occluding the malformed vascular mass. This can be achieved through different approaches: transarterial approach, which directly targets the feeding arteries to deliver embolic agents such as Onyx or NBCA; transvenous approach, used when arterial access is challenging, employing coil-assisted embolization and polymeric embolic agents to manage blood flow; and direct percutaneous puncture, which allows direct access to the nidus for embolization, often used in combination with other methods. Embolic agents used in these procedures include absolute ethanol, which causes extensive endothelial cell damage but carries a high risk of complications; Onyx and NBCA, which are liquid agents that fill the nidus but do not destroy endothelial cells, requiring careful management to prevent recurrence; and mechanical embolization agents, such as coils, which provide mechanical occlusion and reduce flow velocity, aiding in the effectiveness of liquid agents.

In this particular case, the decision to use Onyx glue for the embolization of the posterior tibial artery supplying the AVM was based on the extensive nature of the malformations. Onyx glue was preferred because it can completely obstruct the feeding vessels and prevent the formation of further collaterals. Onyx, an ethylene-vinyl alcohol copolymer dissolved in dimethyl sulfoxide (DMSO), is a nonadhesive liquid embolic agent that solidifies upon contact with blood, creating a cohesive and durable embolus. Its ability to penetrate the complex vascular network of the AVM and provide a more complete and permanent occlusion compared to other agents made it the ideal choice initially. However, significant pain ensued in the medial ankle region after embolization, likely due to ischemia of the posterior tibial nerve or medial plantar nerve. Consequently, Onyx glue was used only once on the posterior tibial artery AVM and not used again due to the resulting local ischemia affecting adjacent nerves.

Subsequent treatments utilized polyvinyl alcohol (PVA) particles and coils. PVA particles were chosen for their ability to occlude small blood vessels effectively, targeting specific areas of the AVM without affecting surrounding tissues. Coils, small spring-like devices made of platinum, were also used. Coils induce clot formation by creating a physical barrier that slows blood flow and promotes thrombosis, effectively blocking the vessel. They are particularly useful for larger vessels or when precise control over the embolization process is required. The large AVM arising from the calf and supplied by the peroneal artery, as well as the AVM in the foot arising from the anterior tibial artery, were treated with these materials.

The interventions in this case were not without complications. The most significant complication occurred after the initial use of Onyx glue, where the patient developed significant pain in the medial ankle region. This pain was attributed to ischemia of the posterior tibial nerve or medial plantar nerve, likely caused by the embolization. To manage this, we administered a local injection of the anesthetic Exparel to alleviate the pain. Given this complication, Onyx glue was not used again, and we opted for alternative embolic materials such as PVA particles and coils. Another potential complication of embolization procedures is the risk of nontarget embolization, where the embolic material inadvertently occludes normal arteries, leading to unintended ischemia and tissue damage. To minimize this risk, we utilized precise imaging techniques and real-time fluoroscopic guidance during the procedures to ensure accurate delivery of the embolic agents. In cases where mechanical embolization agents like coils are used, there is a risk of coil migration or incomplete occlusion, which can result in persistent or recurrent AVM symptoms. In this patient, we carefully selected the size and type of coils to match the specific vascular anatomy and employed multiple imaging follow-ups to confirm the success of the embolization and detect any signs of migration or incomplete occlusion.

Our case involved a 72-year-old female with multiple concurrent AVMs in her lower extremities, presenting a unique and complex challenge. To our knowledge, there are few documented cases of arteriovenous malformations in the extremities, with even fewer specifically involving the foot [4,[14], [15], [16]]. Most documented cases in the foot discuss surgical treatments, and there is limited information available on nonsurgical approaches [16]. One such case describes a 54-year-old woman with a long-standing leg ulcer that was resistant to conventional treatments [17]. It was found that the ulcer was caused by an arteriovenous malformation involving the saphenous vein [17]. The patient underwent successful surgical intervention, which included ligation of the abnormal connections and removal of the affected vein and ulcerated tissue [17]. Yet in our case, treatment of 3 concurrent AVMs was successfully performed endovascularly, and as such, our report sheds light on the efficacy of nonsurgical approaches to AVMs.

Moreover, this case stands out as exceptionally rare due to the simultaneous occurrence of 3 AVMs in a single patient. The presence of 3 concurrent AVMs necessitated an intricate combination of diagnostic and therapeutic strategies to manage her condition effectively. Initial imaging revealed 3 distinct AVMs: the largest malformation arising from the posterior tibial artery contained a small AVF and smaller malformations from the peroneal artery and plantar aspect of the foot. The treatment involved a series of detailed angiograms and embolizations using Onyx glue and Ruby coils. The multi-faceted approach, combining enhanced CT and DSA for precise diagnosis, and the use of various embolization techniques, demonstrated the advanced and tailored care provided to the patient, emphasizing the importance of a comprehensive and adaptable treatment plan in managing complex vascular malformations.

Follow-up imaging and clinical evaluations are critical in monitoring the progression and potential recurrence of AVMs. In our patient's case, follow-up imaging included targeted sonograms and enhanced CT scans, which confirmed residual malformations but no significant arterial flow, indicating successful treatment. Regular follow-ups were scheduled at 3, 6, and 12 months post-treatment, with annual DSA examinations to ensure no relapse and to monitor her condition. The consistent use of advanced imaging techniques during follow-ups allowed us to assess the efficacy of the interventions and adapt the treatment plan as necessary, ensuring optimal patient outcomes.

## Conclusion

Often, arteriovenous malformations (AVMs) are misdiagnosed, leading to incorrect or delayed treatment. Our case emphasizes the need for attentive evaluation in patients presenting with distal lower-extremity ischemia. By integrating detailed imaging, diverse treatment modalities, and thorough follow-up protocols, we successfully managed our patient's AVMs, highlighting the importance of a comprehensive and personalized approach to treating complex vascular aberrations. A multidisciplinary approach is crucial in managing AVMs, involving collaboration among interventional radiologists, vascular surgeons, and other specialists to develop individualized treatment plans that address the unique characteristics of each patient's condition. Currently, there is no standardized protocol for AVM management, emphasizing the need for further clinical research to establish a definitive treatment strategy. Future research should also delve into the genetic and molecular mechanisms underlying AVMs to enhance diagnostic accuracy and therapeutic outcomes.

## Ethics statement

IRB approval was obtained from Providence Medical Center Institutional Review Board.

## Consent for publication

Written informed consent was obtained from the patient for the publication of this report and any accompanying images.

## Data availability

The data that support the findings of this study are available on request from the corresponding author. The data are not publicly available due to privacy or ethical restriction.

## Patient consent

I have obtained written informed consent for publication of this article from the patient.
